# The Value of a Man

**Published:** 1913-04-19

**Authors:** 


### The Hospital

The Workers' Newspaper of Administrative Medicine and Institutional Life, Administration, National Insurance and Health.

No. 1398, Vol. LIV. SATURDAY, APRIL 19, 1913. PRICE ONE PENNY.

### THE VALUE OF A MAN.

It is common knowledge that the assessment of damages in compensation cases is very largely fortuitous. In cases taken into Court it depends primarily on the vagaries of the jury; and these are influenced, or liable to be influenced, by all sorts of factors which should really be excluded altogether, such as class sympathy, ingenious advocacy, and *argumenta ad hominem* of all sorts. In the greater number of cases some form of compromise is arrived at without judicial proceedings; and here, too, the amount accepted as damages fluctuates enormously for accidents of the same extent and consequences. Since no two human beings are precisely alike to begin with, and no two accidental injuries are scientifically provable to be of exactly the same prejudicial effect, it may be argued that an element of variability is inevitable, and that no rigid scheme or table of comparative monetary equivalents for damage sustained is feasible.

Admittedly, the construction of such a table must be extremely complicated, if it is to be of any practical use. But difficulties, after all, exist merely to be overcome; and an ingenious system has been devised in the United States, by Dr. Holt, of Portland, Maine, which is claimed to be a satisfactory solution of them. This branch of physical economics he designates the Value of Man, and it is quite evident that he has put an enormous amount of energy as well as of mathematical and medical ability into the study of that abstruse problem. The tables are extensive, and look at first glance rather like a book of logarithms. A few illustrations of the method of working them will show how they are applied in compensation cases better than any exposition of the root principles, which would necessarily be long and technical.

A shop assistant, aged forty-three, suffers entire loss of hearing in one ear through an accident; but is otherwise not affected. He earns two dollars a day, which fixes his total "economic value," according to Dr. Holt's table, at 10,000 dollars. The damage to the "functional ability" of the body (abbreviated to F) is found by referring to the tables to be 0.12; therefore that which remains is 0.88 of the normal F. The damage to the "competing ability" (C) is much less according to the table. Now the "earning ability" (E) is found by multiplying together the new F and C, and comes out at 0.8641 of the normal. Since the total economic value was originally 10,000 dollars, and is now only 8,641, the diminution of 1,359 represents the loss sustained. To this has to be added such items as pain and suffering, medical expenses, loss of time during recovery, and so on; and the result shows the total compensation to which the man is entitled.

A rather more complicated case will show how Dr. Holt's system is prepared to deal with more involved injuries. Suppose the same shop assistant suffered total loss of sight in one eye, total loss of hearing in one ear, and loss of the left hand at the wrist joint. The loss of F for the amputation is 0.20; for the loss of sight 0.18; for that of hearing 0.12. Now sight and hearing are of a similar order, and the table requires that the coefficients of loss be added, not multiplied, together. Therefore the total loss of F for these two is 0.30. Thus, for the amputation alone F=0.80, and for the eye and ear alone F=0.70. Not being of the same order, these coefficients have to be multiplied; the total of F after the accident is therefore 0.56 of the normal. Now in this case the competing ability is held to be damaged more than F, and is reckoned as equal to the square of the latter. Thus $E = C \times F = 0.56^2 \times 0.56 = 0.1756$. In other words, this man has lost 8,244 dollars of his economic value of 10,000.

It would be easy to point out omissions in the tables, objections to the uniform system of rating everyone on the basis of his actual salary at the moment, failure to take into account the differing exigencies of different occupations, and many other points which Courts of law or arbitration rightly consider in fixing compensation. But, in any case, Dr. Holt claims no infallibility for his tables, and admits that some of them may have to be altered.

And it is more to the point to acknowledge that his attempt to adjust these intricate problems of economics on a rational scientific basis is well conceived and honestly carried out. Even loss of taste is provided for in the tables: we may note, parenthetically, that for total loss of taste the allowance is 0.06 of F, which on the face of it seems excessive for anyone except a tea-blender or a wine-merchant. And these exceptional cases would in any case be allowed for by adjustment of the value of other factors in the calculation.

The difficulty about the comparative importance of various organs in different occupations is got over, we presume, by the allowance made with regard to C, the "competing ability." It seems to be a matter of discretion in working this out, how much this function shall be held to be damaged as compared with F. But if this is left to the discretion, it becomes a matter of argument; doubtless the employee would estimate the damage to C on a much more liberal table (Dr. Holt provides seven different ones to choose from) than the employer would. Herein lies plentiful opportunity for skilful advocacy; so Dr. Holt's invention is not likely to affect the earnings of barristers, even if it were adopted. We ought to add that in all skilled professions an extra factor, T ("technical ability"), is brought into the arithmetic; and this again provides plenty of scope for differences of opinion and litigation based upon them.

